# An infected chicken kidney cell co-culture ELISpot for enhanced detection of T cell responses to avian influenza and vaccination

**DOI:** 10.1016/j.jim.2014.10.012

**Published:** 2015-01

**Authors:** Raul Ruiz-Hernandez, Marylene Peroval, Amy Boyd, Devanand Balkissoon, Karen Staines, Adrian Smith, Colin Butter

**Affiliations:** a The Pirbright Institute, Compton Laboratory, Compton, Newbury, Berkshire RG20 7NN, UK; b University of Oxford, Department of Zoology, South Parks Road, Oxford OX1 3PS, UK

**Keywords:** ChIFNγ, Chicken interferon-gamma, APC, Antigen presenting cells, MHC, Major histocompatibility complex, CTL, Cytotoxic T lymphocytes, rFPV, Recombinant Fowlpox virus, MVA, Modified Vaccinia virus Ankara, CKC, Primary chicken kidney cells, Adaptive antiviral responses, Antigen presentation, Avian influenza, IFNγ ELISpot, CD8 T cell

## Abstract

A better understanding of the immune responses of chickens to the influenza virus is essential for the development of new strategies of vaccination and control. We have developed a method incorporating infected chicken kidney cells (CKC) in culture with splenocytes in an IFNγ ELISpot assay to enumerate ex vivo responses against influenza virus antigens. Splenocytes from birds challenged with influenza showed specific responses to the influenza virus, with responding cells being mainly CD8 positive. The utility of the assay was also demonstrated in the detection of an antigen specific enhancement of IFNγ producing cells from birds vaccinated with recombinant Fowlpox vectored influenza nucleoprotein and matrix protein.

## Highlights

• Chickens infected with avian influenza developed IFNγ responses.
• The use of infected CKC in ELISpot overcomes limitations at detection of responses.
• This methods allows the quantification of influenza specific CD8 T cells.
• The use of recombinant virus to infect CKC can further define antigen specificity.

## 1 Introduction

Avian influenza viruses (AIVs) belong to the Orthomyxoviridae family and are classified according to their haemagglutinin (HA) and neuraminidase (NA) proteins. On the basis of their ability to cause disease in poultry, avian influenza viruses are further classified as low pathogenic (LPAI) and highly pathogenic (HPAI), both causing severe financial losses to the poultry industry. Poultry also act as a reservoir for AIVs and thus provide an environment for the emergence of novel AIV subtypes, which may present a threat to human health, through the processes of recombination and re-assortment. Hence, improved understanding of influenza virus infections in chickens is an important aspect of developing new control measures, including vaccines for use in poultry. Improved control of influenza in chickens will protect the poultry industry and reduce the risk of zoonotic transfer to humans.

Although influenza viruses are endemic in avian species (Stech et al., 2009) understanding of influenza-specific cellular responses is more limited in chickens than in humans or mice; until recently a paucity of reagents and techniques has impeded a comprehensive study in birds. Although most studies of host responses to influenza infection or vaccination in birds have focused on the production of neutralizing antibodies, it is clear that cell mediated immunity (CMI) is also relevant (Suarez and Schultz-Cherry, 2000). The principal route for the presentation of viral antigenic peptides involves the major histocompatibility complex I (MHC I) pathway and results in antigen presentation to CD8^+^ T cells (Subbarao and Joseph, 2007). In birds and mammals, influenza-specific CD8^+^ cytotoxic cells become activated and produce IFNγ during infection in response to the engagement of their T cell receptors with influenza-derived peptides in the context of MHCI on the surface of antigen presenting cells (APC) (Rock et al., 1990; Suarez and Schultz-Cherry, 2000; Novak et al., 2001; Subbarao and Joseph, 2007). Cytotoxic T cell responses can be generated against a variety of influenza proteins including surface associated HA and NA antigens as well as internal proteins such as matrix protein (M1) and nucleoprotein (NP). These CD8^+^ T cell responses contribute to the control of influenza virus replication within cells, thereby enabling viral clearance and limiting viral spread (Suarez and Schultz-Cherry, 2000; Kwon et al., 2008). A suppression of these responses may contribute to high and disseminated viral replication in chickens, contributing to the pathogenicity of LPAI viruses (Kwon et al., 2008). Enhancement of CTL responses (principally against the most conserved epitopes) has thus become the aim of vaccines designed to produce heterosubtypic immunity to influenza in humans (Berthoud et al., 2011) and chickens (Boyd et al., 2012).

A number of techniques and reagents are currently in use for the study of T cell responses in poultry. These include the measurement of antigen specific proliferation by flow cytometry (Dalgaard et al., 2010) intracellular cytokine staining (De Boever et al., 2010), measurement of IFNγ production by Enzyme-Linked Immunosorbent Assay (ELISA) (Ariaans et al., 2009; Rauw et al., 2011) and Enzyme-Linked Immunosorbent Spot (ELISpot) assay (Ariaans et al., 2008, 2009). CTL responses to infectious bronchitis virus have previously been monitored using MHC matched chicken kidney cells (CKC) serving as antigen presenting cells (APC) (Seo and Collisson, 1997). Through the use of MHC matched infected cells as surrogate APC, the measurement of chicken IFNγ responses against whole influenza virus or viral proteins has been achieved using an indirect method based on the ability of IFNγ to activate the HD11 macrophage cell line (Gobel et al., 2003; Singh et al., 2010a,b).

Peptides are often used to study antigen specific responses, and this method has been applied successfully in birds (Haghighi et al., 2009; Reemers et al., 2012). While the use of peptide libraries to identify influenza antigen specific responses can be exquisitely informative, it also has his limitations. The cost of peptide libraries can be prohibitive for many labs, even before technical considerations. The use of a library of predicted binding peptides excludes epitopes that are not predicted due to an incomplete understanding of the binding motifs of chicken class I MHC. This may be particularly challenging with haplotypes such as B21 that has a highly promiscuous motif (Koch et al., 2007). Although peptide length and motif can give an indication of which group of cells responds, this does not provide definitive information regarding the phenotypic identification of effector T cells (CD4 versus CD8), and there are no significant data regarding processes such as cross presentation in poultry model, rendering interpretation of peptide data difficult. In addition, techniques such as intracellular staining are technically challenging, requiring many manipulations of the cells. ELISpot, while sensitive, provides no information as to the effector cell phenotype unless the responding cells are sorted prior to plating. This is also true for the HD11 activation method, which requires culture and stimulation of HD11 as an extra step.

In the present study we set out to develop a method to preferentially detect CD8 T cell responses. We hypothesized that by infecting cells which only express class I MHC with AIVs and culturing these with splenocytes from infected birds we would potentiate detection of influenza specific CD8^+^ T cells. We first derived a chicken kidney cell line from inbred chickens MHC matched to our experimental cohort, tested that the CKC expressed MHCI but not MHCII, then infected them with influenza virus. After irradiation these cells were co-cultured in ELISpots with MHC matched splenocytes either from chickens exposed to influenza virus or from vaccinated chickens. In both experimental scenarios we were able to demonstrate the presence of antigen specific T cells. We also demonstrated by flow cytometry that the IFNγ producing cells were principally CD8 positive. The assay was reproducible, with high sensitivity and low background noise, and will be a useful tool in the analysis of CD8 T cell responses.

## 2 Material and methods

### 2.1 Animals

Inbred lines of White Leghorn chickens, Line O (haplotype B21) or Line 15 (B15) (Miller et al., 2004), were produced and maintained at the Pirbright Institute (Compton, UK) in specific pathogen-free (SPF) conditions and fed ad libitum. For infection studies birds were housed in self-contained BioFlex® B50 Rigid Body Poultry isolators (Bell Isolation Systems). Animal procedures were carried out in accordance with local ethical review and UK Home Office requirements (Home Office, 1986).

### 2.2 Viral stocks

LPAI virus (A/Turkey/England/1977/H7N7) was grown in embryonated chicken eggs using standard methods described elsewhere (World Health Organization. Dept. of Epidemic and Pandemic Alert and Response., 2002). Viral titer was estimated by plaque assay on Madin-Darby canine kidney (MDCK) cells, using standard techniques (Gaush and Smith, 1968).

Virus was inactivated in a final concentration of 0.094% β-propiolactone (ACROS Organics, Geel, Belgium), as described previously (Jonges et al., 2010) and aliquots were stored at − 80 °C until its use. Inactivation was verified by the absence of plaques on MDCK cells. Recombinant Fowlpox virus (rFPV) vectors expressing NP and M1 transgenes from avian influenza A/Turkey/Turkey/1/2005 (H5N1) or GFP were the kind gift of Dr. Mike Skinner (Imperial College). Modified Vaccinia Ankara (MVA) virus expressing a fusion protein of nucleoprotein and matrix protein 1 (MVA-NpM1) from influenza A/Panama/2007/99 (H3N2) was supplied by the Vector Core Facility at the Jenner Institute (Oxford, UK) (Berthoud et al., 2011).

### 2.3 Vaccination and infection

In a first round of experiments, 3 week old birds were randomly allocated to infected or control groups. Birds were challenged by intranasal inoculation of LPAI (A/Turkey/England/1977 H7N7) at a dose of 3.4×10^7^ pfu in 100 μl PBS per bird. In the second round of experiments, birds were vaccinated subcutaneously with 10^5^ pfu rFPV at 1 day old, boosted with the same dose at 9 days old, and challenged with LPAI, as above, at 4 weeks old. Birds were killed 10 days post-infection.

### 2.4 Measurement of viral shedding

Sterile polyester tipped swabs (Fisher Scientific, UK) were used to sample buccal cavities, transferred to a solution of viral transport media (World Health Organization. Dept. of Epidemic and Pandemic Alert and Response., 2002), vortexed briefly, clarified of debris by centrifugation at 450 ×*g* for 2 min and stored at –80 °C. RNA was extracted using the QIAamp One-For-All Nucleic Acid Kit on an automated extraction platform (Qiagen BioRobot Universal System, Qiagen, UK) according to the manufacturer's instructions. Quantitation of influenza virus in RNA from swabs was performed by analysis of matrix gene transcripts. A single step real-time reverse transcriptase PCR was carried out using the Superscript III Platinum One-Step qRT-PCR Kit (Life Technologies, UK). Primers and a probe specific for a conserved region of the Influenza A Matrix gene were used as described previously (Spackman et al., 2002). Cycling conditions were: 50 °C, 5 min; 95 °C, 2 min; and then 40 cycles of 95 °C, 3 s and 60 °C, 30 s, using a 7500 fast real-time PCR machine (Applied Biosystems, UK). Results are expressed in terms of the threshold cycle value (Ct), the cycle at which the change in the reporter dye signal passes a significance threshold (Rn).

### 2.5 Cell lines

MDCK cells were grown in Dulbecco's Modified Eagles Medium (DMEM) with Glutamax (Life Technologies), supplemented with non-essential Amino Acids (Sigma), 100 U/ml penicillin, 100 μg/ml streptomycin and 10% fetal bovine serum (FCS). Chinese hamster ovary (CHO) cells were grown in Ham's F12 medium (Life Technologies) with 10% FCS. Puromycin HCl (Enzo) was used at 20 μg/ml for selection of IFNγ transfected lines and at 15 μg/ml for maintenance of transfected CHO cells. Cell cultures were maintained in 5% CO_2_ at 37 °C.

Primary chicken kidney cell (CKC) lines were established from 10 day old birds following guidelines previously described (Seo and Collisson, 1997). Briefly, cells were dispersed with trypsin digestion and cultured in 150 or 75 cm^2^ tissue culture flasks. The CKC adherent cells were continuously cultured by passage every 4–6 days in Minimum Essential Medium (MEM) supplemented with tryptose phosphate broth (TPB), glutamine, 1M HEPES, fungizone, 100 U/ml penicillin, 100 μg/ml streptomycin and 10% FCS. Chicken cell cultures were maintained in 5% CO_2_ at 41 °C.

### 2.6 Generation of anti-chicken IFNγ monoclonal antibodies

Antibodies were generated using a technique previously described (Staines et al., 2013). Briefly, chicken IFNγ was amplified from a spleen cDNA library using the following primers; IFN-Foward-NheI (5′-AGCCATCAGCTAGCAGATGACTTG) and IFN-Reverse-BglII (5′-ATCTCCTCAGATCTTGGCTCCTTTTC) and cloned into an Ig-fusion protein vector. To obtain ChIFNγ monoclonal antibodies, we immunized mice with two intramuscular injections of 100 μg of the IFNγ-IgG1Fc plasmid diluted in PBS (endotoxin free, Qiagen Endofree Plasmid Maxi Kit) at four week intervals. After a further four weeks, mice received a final boost with an intraperitoneal injection of 50 μg purified fusion protein and were sacrificed four days later for preparation of splenocytes which were fused with NS0 hybridoma partner cells using established methods. Hybridoma supernatants were first screened by ELISA for antibodies binding fusion protein immobilized with anti-human IgG and detected with HRP conjugated goat anti-mouse IgG. Antibodies recognizing the human Ig moiety of the fusion protein were eliminated by a similar ELISA using a control fusion protein containing the same human IgG1 sequence (DEC205-IgG1Fc, (Staines et al., 2013)). Antibodies from two IFNγ-specific clones, AF10 and EH9, were purified from high density culture (miniPERM, Sarstedt) with Hi Trap Protein G HP columns (Amersham-Pharmacia, UK) according to the manufacturer's instructions. After dialysis against PBS, the concentration of these antibodies was estimated by measurement of the absorbance at 280 nm.

### 2.7 Preparation of infected CKC

CKC were infected with A/Turkey/England/1977/H7N7 for use in co-culture as previously described (Singh et al., 2010a). Briefly, confluent monolayers of CKC (after a minimum of 8 passages) were infected with AIVs for 1 h at a Multiplicity of Infection (MOI) of 3–5, washed with PBS, and incubated for 4 h with CKC growth media without FCS, supplemented with TPCK trypsin (Sigma). Cells were then washed, dispersed with trypsin, washed again, counted, resuspended in leukocyte culture media and then irradiated with 3000 rad using a Gammacell 1000 Elite caesium 137 gamma irradiator (Nordion, Canada).

For infection with recombinant MVA, CKCs were infected by incubation for 1 h at 37 °C at an MOI of 5. We optimized these conditions through analysis of GFP transgene expression by confocal microscopy (Supplementary Fig. 1). Following incubation, cells were washed, counted, irradiated as described, and resuspended in leukocyte culture media. The irradiated CKC were used at a ratio of 1:10 (CKC:splenocyte) in co-culture ELISpot.

For confocal imaging 5×10^4^ primary CKC in growth media per chamber of an 8 chamber slide (Lab-TekII, Nunc) were incubated at 41 °C, 5% CO_2_, for 1 day. Any non-adherent cells were discarded and the adherent cell population was infected with MVA-GFP constructs as described above. After incubation, cells were fixed with a solution of 4% paraformaldehyde for 20 min, and then washed in PBS. Nuclei were stained by incubation with 2 μg/ml DAPI (Sigma) for 10 min. Sections were mounted in Vectashield (Vector Laboratories) and analyzed using a confocal microscope (Leica SP2 with 405-, 488-, and 568-nm lasers).

### 2.8 IFNγ ELISpot

Spleens were macerated in cold sterile PBS and passed through a 100 μm cell strainer (Fisher, UK). Cell suspensions were centrifuged at 220 × *g* for 10 min at 4 °C and resuspended in culture media (RPMI 1640 medium with Glutamax supplemented with 10% FCS, 100 U/ml penicillin, and 100 μg/ml streptomycin) (all from Life Technologies, UK) before under-laying Histopaque 1119 (Sigma, UK) and centrifuged at 2000 rpm (492 ×*g*) for 20 min at 4 °C. Cells harvested from the interphase were washed twice, counted using a Countess™ automated cell counter (Life Technologies) and resuspended at 5 × 10^6^/ml. ChIFNγ ELISpot was carried out as described previously (Ariaans et al., 2008), using either antibodies from a commercially available kit for detection of chicken IFNγ protein (chicken IFNγ ELISA kit, Life Technologies ®) or EH9/AF10 antibodies produced as described. 96-well plates (MAIPS4510, Millipore) were coated overnight at 4 °C with the commercial capture antibody or EH9, diluted in pH 9.6 50 mM carbonate/bicarbonate buffer at a final concentration of 2 μg/ml, and washed with PBS + 0.1% Tween 20 at this stage and between all subsequent steps. Plates were blocked with experiment culture media. Chicken leukocyte suspensions consisting of 3 or 5 × 10^5^ cell/well were maintained in 5% CO_2_ at 41 °C for 1 or 2 days. Cells were incubated in the presence of either culture medium or medium supplemented with one of the following stimuli to a final volume of 200 μl per well: phorbol 12-myristate 13-acetate (PMA 500 ng/ml) plus ionomycin (750 ng/ml, Sigma); Concanavalin A (ConA, 10 μg/ml final; Sigma); inactivated or live virus (MOI 3-5); prepared exogenous APCs (1:10, CKC: splenocyte), pooled or individual peptide (5 μM). A library of 62 overlapping peptides spanning NP protein from A/Turkey/England/1977/H7N7 virus (challenge virus) was synthesized commercially (Neobioscience, Massachusetts, USA), resuspended in DMSO or in a solution of 50% acetic acid in water, aliquots stored at − 20 °C until use, and then diluted to a final concentration of 5 μM in culture wells. Peptides were 18 aa long and 10 aa overlapping (Supplementary Table 1). When the chicken IFNγ ELISA kit (Life Technologies®) was used, ELISpot plates were then incubated at RT with the biotinylated detection antibody (1 μg/ml) followed by an incubation with streptavidin-horseradish peroxidase conjugate at a 1/2000 dilution. Otherwise plates were incubated with the detection antibody AF10, followed by an incubation with first a biotinylated goat anti-mouse anti isotype IgG2b (AF10 isotype) antibody (Southern biotech) followed by avidin-HRP (Southern Biotech). Plates were developed by incubation with 100 μl per well of 3-amino-9-ethylcarbazole, (AEC, Merck Chemicals, UK). After spot development, plates were rinsed with tap water and allowed to dry overnight before counting using an ELISpot plate reader (AID systems, Germany). Results were expressed as number of spots (SFU, spot forming unit) per 10^6^ splenocytes. Depending on the stimuli used, experiments were carried out in triplicate (whole virus or CKCs) or in duplicate (peptides).

### 2.9 Serum sample processing and hemagglutination inhibition assays

Blood samples (0.5–1 ml/bird) from all challenged birds were drawn from a wing vein 2 weeks after infection to evaluate humoral responses against influenza virus. These were left to clot at room temperature (RT) and sera were retrieved after centrifugation and stored at − 20 °C until analysis. A standard HI test was used to measure serum AIV antibody titers, which were expressed in log2 mean HI titers in each sample for each group (Spackman, 2008).

### 2.10 Flow cytometry and intracellular staining of ChIFNγ

Cultured cells were resuspended in U bottom 96-well plates in FACS buffer (PBS containing 1.0% BSA and 0.1% sodium azide) and incubated with normal mouse serum (1%) for 10 min at RT to block non-specific binding. For surface staining, cells were incubated, protected from light, for 15 min at room temperature with appropriate dilutions of antibodies: mouse anti-chicken CD4-FITC (CT4) (Southern Biotech), CD8α-PerCp (Tregaskes et al., 1995). After incubation, cells were washed twice with FACS buffer and were either used for intracellular staining or fixed with a solution of 2% paraformaldehyde in PBS. Incubation with primary antibodies to MHC I (Salomonsen et al., 1987) and MHC II (Kaufman et al., 1990) was followed by Alexa-647 conjugated goat anti-mouse antibody (Life Technologies). Secondary antibody alone or unconjugated goat anti-mouse antibody (Life Technologies) was used as an unstained control for surface MHC staining. Intracellular staining was carried out as described previously (Ariaans et al., 2008). Briefly, splenocytes from challenged birds or non-infected controls were seeded in a 96-well round-bottom plates (Nunc) at 10^6^ cells/well in a final volume of 200 μl of culture media or culture media supplemented with the different stimuli at the concentration described in the ELISpot technique (except PMA which was used at 50 ng/ml). Cells were cultured using the conditions described above for ELISpot assays (24 h culture). For intracellular staining, during the last 2 h of culture, cells were treated with Brefeldin A according to the manufacturer's instructions (Cytofix/Cytoperm™ Plus Fixation/Permeabilization kit, BD Biosciences). To avoid non-specific binding signal, we preincubated cells with Cytofix/Cytoperm™ buffer containing 2% normal mouse serum and further staining steps involved Cytofix/Cytoperm™ washing buffer containing 1% normal mouse serum (Biosource). To confirm the specificity of the anti-IFNγ antibody EH9 we also employed a validated anti-IFNγ [mAb80 (Ariaans et al., 2008)]. Purified fractions of both antibodies were conjugated using Alexa Fluor® 647 monoclonal antibody labeling kit (Molecular Probes) according to the manufacturer's instructions. A mouse isotype matched control antibody IgG1 Alexa Fluor® 647 (Life Technologies) was employed at the same concentration as EH9 and Mab80. For analysis, a gate on the FSC/SSC region of lymphocytes was selected and a minimum of 10,000 events were acquired on a FACSCalibur instrument using Cell Quest software (BD Becton Dickinson). Flow cytometry analysis indicates that non-adherent CKC were not present at significant levels (data not shown). FlowJo software (TreeStar) was used to analyze flow cytometry data.

### 2.11 Statistical analysis

A paired or unpaired t-student test or one-way ANOVA was performed using GraphPad Prism (version 6.0 for Windows, GraphPad Software, San Diego, California, USA).

## 3 Results

### 3.1 Validation of anti-chicken interferon gamma antibodies

Screening identified two anti-chicken IFNγ antibodies (clones EH9 and AF10) which were shown by ELISA to bind recombinant chicken IFNγ and to work effectively as an antibody pair in capture ELISA (Supplementary Fig. 2A–C). We subsequently compared this antibody pair with commercially available antibodies [from Life Technologies (Ariaans et al., 2008; Reemers et al., 2012)] in ELISpot assays. There were no differences in the numbers of spots from the same samples of chicken splenocytes stimulated with Con A using either antibody pairs (EH9/AF10: 338.1 ± 23.2 SFU/10^6^ cells; Life Technologies: 338.9 ± 21.3 SFU/10^6^ cells, n = 9 (Supplementary Fig. 2D)).

### 3.2 Viral shedding dynamics and immune responses

**Fig. 1:**
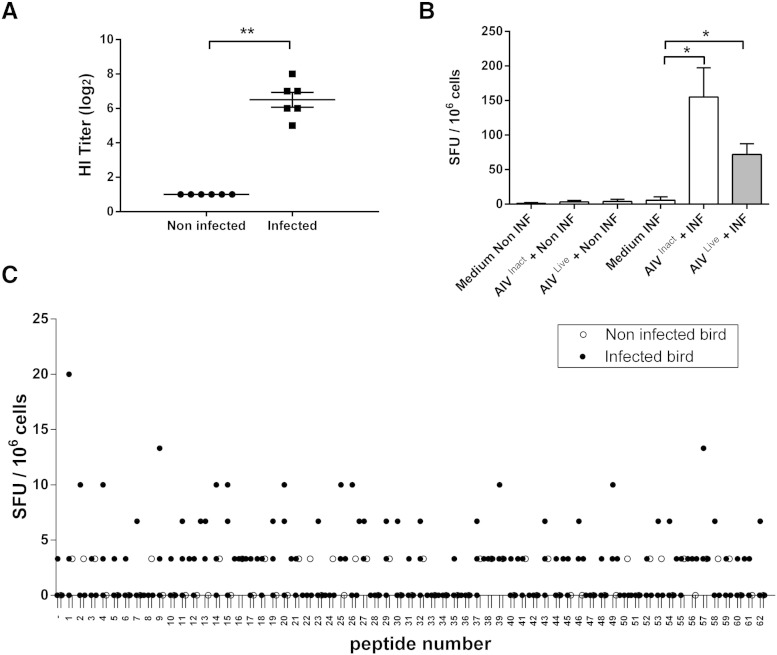
Viral shedding, serum antibody  titers and ELISpot responses of challenged birds. Birds were challenged with LPAI influenza strain H7N7/turkey/Eng/77. A) Influenza antiserum titers, HI assay from non-infected birds and infected birds; results expressed as mean ± S.E.M. (n = 6 per group). B) ChIFNγ ELISpot responses of splenocytes from infected birds (INF, n = 4) sacrificed 2 weeks after challenge and non-infected birds (non-INF, n = 3) were cultured with neutralized influenza virus (AIV^Inact^) and live virus (AIV^Live^). Results expressed as mean (± S.E.M.) of spot forming units (SFU) per million cells. Significance of data is represented as follows: * = p < 0.05, ** = p < 0.01. SFU: spot forming units. C) ChIFNγ ELISpot responses of peptide library against influenza viral nucleoprotein (NP). In total, 62 different peptides were analyzed in splenocytes from individual birds infected at 2 weeks post-challenge (black dot, n = 3) and control not infected (white circle, n = 1).

Following intranasal infection of Line O birds with LPAI H7N7, buccal swab samples were analyzed for the presence of influenza M1 transcript by qRT-PCR. These were found to be positive from the earliest sampling time point at day 4 post-infection. Viral transcript was still detectable albeit at a lower level (p < 0.05) in the buccal swabs at day 6, and was undetectable at day 10 (data not shown). Challenged birds exhibited significantly higher HI titers compared to non-infected controls (Fig. 1A, p < 0.01). All subsequent experiments were performed in Line O birds, with the exception of the vaccine cohort (Line 15, final figure).

We tested our antibody pair for use in ELISpot with live or beta propiolactone inactivated challenge-strain virus to stimulate splenocyte responses (Fig. 1B). Splenocytes from control (non-infected) birds did not produce IFNγ when exposed to either live or inactivated virus. In contrast, splenocytes from infected birds did produce IFNγ (p < 0.05) following exposure to both live (72.0 ± 15.4 SFU/10^6^ cells) and inactivated virus (155.2 ± 42.3 SFU/10^6^ cells), as expected. The use of live virus consistently yielded lower responses than the use of inactivated virus in all samples, although this difference was not statistically significant.

To identify epitope-specific responses, we employed an NP peptide library corresponding to the challenge virus. We analyzed responses to pooled peptides at 1 week post-infection (Supplementary Fig. 3) and to individual peptides 2 weeks after infection (Fig. 1C). Responses to individual peptides were low, not consistent between birds, and not statistically significantly different between control and infected birds. In the following experiments, an alternative strategy to detect specific IFN responses was developed.

### 3.3 CKCs express only MHC class I following repeated passage

**Fig. 2:**
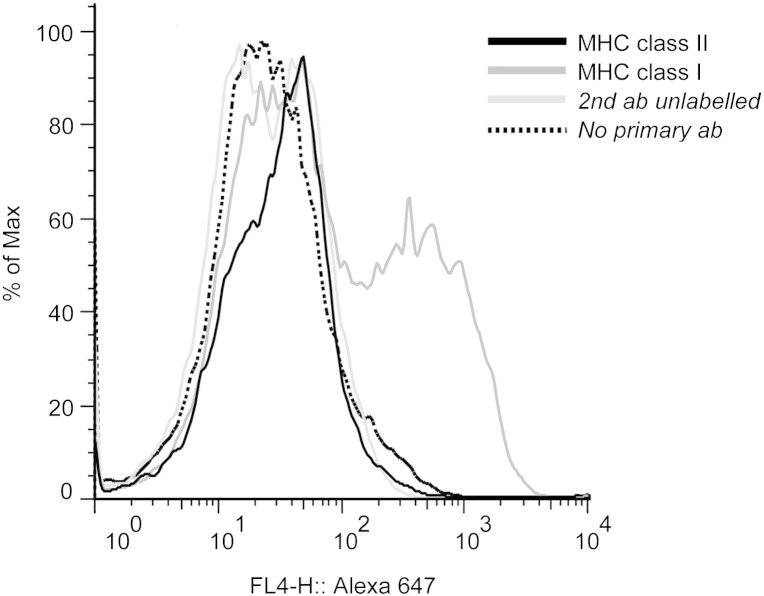
Analysis of MHC class I and II expression on CKC passaged at least 8 times. Expression of MHC classes I and II on CKC detected using an Alexa 647 labeled secondary antibody. Dark line = class II, dark gray line = class I, light gray line = unlabelled secondary antibody, dashed line = only Alexa 647 labeled secondary antibody.

To potentiate the detection of influenza-specific CD8 T cell responses, we generated a CKC cell line expressing only MHC class I. We passaged CKC from Line O birds a minimum of eight times. We then analyzed the cells by flow cytometry for the expression of MHC classes I and II. The passaged CKC were found to exclusively express MHC class I (Fig. 2).

### 3.4 ELISpot using infected CKC co-culture method

**Fig. 3:**
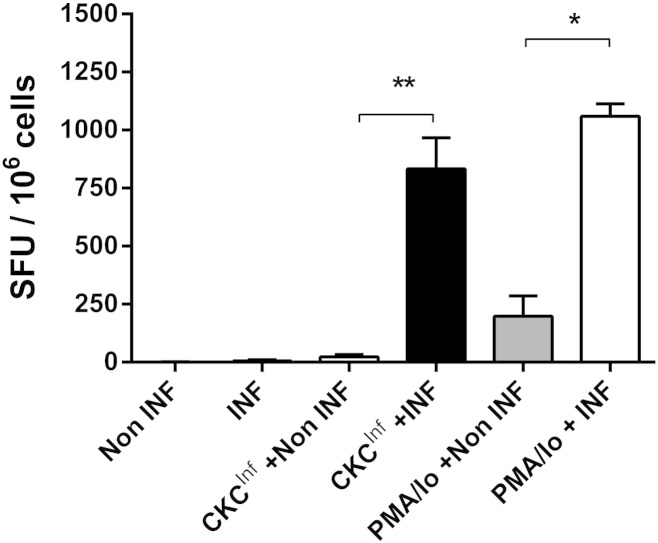
Splenocyte IFNγ co-culture ELISpot responses to infected CKC. Splenocytes from infected birds (INF, n = 4) sacrificed 2 weeks after challenge and control non-infected birds (non-INF, n = 3) were cultured with infected chicken kidney cells (CKC^INF^). Results expressed as mean (± S.E.M.) of spot forming units (SFU) per million cells. Significance of the data is represented as follows: * = p < 0.05, ** = p < 0.01. SFU: spot forming units.

Having validated the necessary individual components we introduced the method of co-culture of responding cells with infected CKCs. Despite the fact that so many antigen specific cells were detectable in co-culture with infected CKCs, the background response for this assay was extremely low (control and INFγ only data, Fig. 3), demonstrating its specificity and sensitivity. Splenocytes from infected birds (2 weeks post-infection) produced extremely high (mean: 833 ± 134 SFU/10^6^ cells) numbers of spot forming units when co-cultured with infected CKC (Fig. 3). This response was significantly different (p < 0.01) from the response seen with infected CKC co-cultured with splenocytes from non-infected (control) birds. The SFU count seen with co-culture of infected CKC with infected splenocytes was close to that seen with cells from infected birds stimulated with PMA/ionomycin (1060 ± 53 SPU/10^6^ cells), suggesting that antigen specific antiviral IFNγ producing cells constitute the majority of those able to rapidly produce IFNγ. It was interesting to note that splenocytes from infected birds have greater SFU responses to PMA in our study (discussed below).

### 3.5 Analysis of IFNγ positive cells from co-culture assay

**Fig. 4:**
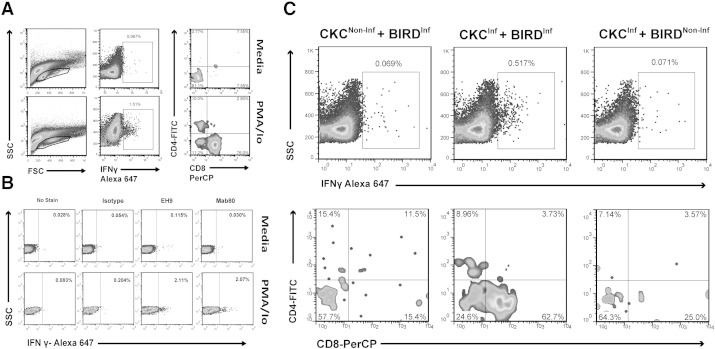
Intracellular IFNγ staining and phenotype of responding lymphocytes following co-culture. A) Representative example of gating of live lymphocytes in intracellular staining. Lymphocyte gate was designated for the region of lymphocytes stimulated with PMA/Io (bottom left figure) and applied to other culture conditions (media alone, upper left figure). B) Comparison of EH9 (in house) and mAb80 (previously described) intracellular staining for IFNγ; both antibodies detected a similar percentage of IFNγ^+^ cells in media control or PMA/ionomycin stimulated splenocytes. Matched IgG isotype control was also employed. C) Representative example of intracellular staining of CKC co-cultured with splenocytes from an infected bird (BIRD^INF^) or control non-infected bird (BIRD^NON-INF^): upper row figures, percentage of IFNγ positive cells following co-culture with non-infected (CKC^non-Inf^) or infected (CKC^Inf^) CKC; lower row figures, CD4^+^ and CD8^+^ lymphocyte populations in the IFNγ positive populations from above figures.

To analyze the phenotype of the responding splenocytes from infected birds we performed intracellular staining on cells from co-culture assays. We first validated antibody (EH9) against a previously published anti IFNγ antibody (mAb80, (Ariaans et al., 2008)) using IFNγ transfected CHO cell lines (Supplementary Fig. 4) and in splenocytes stimulated with PMA/ionomycin (Fig. 4A). There was no statistically significant difference between results obtained with the two antibodies. Non-specific signal was not detected by isotype control staining (Fig. 4B).

We then analyzed the phenotype of IFNγ expressing cells from infected birds, following co-culture with either infected or non-infected CKC. Data shown are for a representative sample from infected and non-infected birds (Fig. 4C) gating in the same FSC/SSC lymphocyte region (Fig. 4A) for all conditions. The greatest number of interferon gamma producing cells was detected during co-culture of infected CKC with splenocytes from infected birds (0.517%), compared with splenocytes from infected birds co-cultured with non-infected CKC (0.069%), and splenocytes from non-infected birds co-cultured with infected CKC (0.071%). It is important to note that the majority of IFNγ positive splenocytes from infected birds co-cultured with infected CKC were CD8 positive (> 60%, Fig. 4C).

### 3.6 Detection of antigen specific IFNγ responses in splenocytes from recombinant Fowlpox vaccinated birds employing CKC infected with recombinant virus MVAGFP/MVANpM1 after influenza challenge

Having established the utility of the co-culture ELISpot we used the technique to analyze influenza antigen specific responses in birds vaccinated (prime and boost) with recombinant Fowlpox (F9) or recombinant Fowlpox-NpM1 (F9-NpM1), and then challenged with an influenza virus with heterologous nucleoprotein and matrix protein. Instead of infecting the CKC with influenza virus we used recombinant MVA carrying either a GFP or NpM1 fusion transgene (homologous to the Fowlpox recombinant) then irradiated the infected CKC as described.

**Fig. 5:**
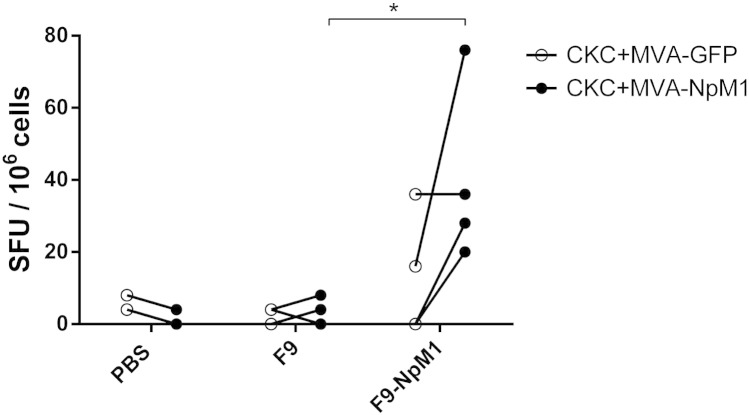
IFNγ co-culture ELISpot using CKC infected with recombinant MVA and splenocytes from birds challenged with LPAI. ELISpot responses in splenocytes from infected birds from unvaccinated (PBS group, n = 3) or birds vaccinated with a recombinant vaccine Fowlpox (F9 group, n = 4), or Fowlpox^NpM1^ (F9-NpM1 group, n = 4). Splenocytes were cultured with either irradiated MVA^GFP^ infected CKC (black dot) or irradiated MVA^NpM1^ (white circle) infected CKC. Lines indicate samples from the same spleen under the two previous conditions. Results were expressed as spot forming units (SFU) per million cells. Significance of the data is represented as follows: * = p < 0.05.

Three of the four F9-NpM1 vaccinated birds challenged with influenza showed IFNγ responses that distinguished them from F9 vaccinated and challenged birds (Fig. 5) (40.0 ± 12.5 vs. 3.0 ± 1.9, p < 0.05). The majority of responses in the F9-NpM1 vaccinated birds were greater with CKC infected with MVA-NpM1 fusion transgene. Some responses were also observed with F9-NpM1 vaccinated birds when APCs were infected with MVA-GFP (although this result was not significant). Our results with vaccinated birds thus suggest that this technique can be used to evaluate vaccine induced responses, and additionally that recombinant viruses expressing appropriate transgenes can be used to replace influenza infection of CKC. This permits the analysis of more defined antigen specific responses while reducing the requirement to handle live influenza virus in the laboratory.

## 4 Discussion

We have developed a method to potentiate the detection and analysis of influenza antigen specific T cells utilizing infected CKC to present viral peptides in a manner biologically relevant to CD8 T cells. We have demonstrated that our co-culture ELISpot detects greater numbers of antigen specific CD8 T cells than ELISpot with whole virus as an antigen. Our assay can also be adapted to use recombinant viruses to infect CKC, increasing its specificity and reducing the requirement to work with live influenza virus. Our results are the first to demonstrate detection by flow cytometry of influenza-specific IFNγ responses in individual T cells from LPAI infected birds.

The ability of our method to detect such large numbers of antigen specific T cells (similar numbers to positive controls with PMA/ionomycin, see example Supplementary Fig. 5) likely reflects not only the high promiscuity of the B21 haplotype, but also the fact that our CKC cell line expresses only MHC class I and presents peptides following a biologically relevant infection process.

In ELISpot using whole influenza virus we were able to detect antigen specific responses, although these were much lower (Fig. 1). Although ELISpot has previously been used to measure antiviral responses against other avian viruses, including NDV (Ariaans et al., 2008) and IBV (Ariaans et al., 2009), it has never been employed to analyze avian responses to influenza. In the present study, following challenge with H7N7 LPAI, the birds became serologically positive and showed specific IFNγ responses, irrespective of whether inactivated or live avian influenza virus was added to endogenous APCs (Fig. 1). Additionally, ELISpot with live virus added to splenocytes from infected birds further reduced SFU counts. It is possible that live virus affects the interactions, and/or the functionality, of cells in vitro (Hinshaw et al., 1994; Oh et al., 2000; Hao et al., 2008).

It was interesting to note that splenocytes from infected birds have greater SFU responses to PMA in our study. PMA does not activate all T cells (Suzawa et al., 1984; Kim et al., 1986)., It may be that antigen experienced cells (from infected birds) have a lower threshold of activation and are activated more readily by PMA, hence the higher SFU counts in the infected cohort positive control compared with the non-infected. Another possibility is altered lymphocyte subset frequencies in infected birds. We did not note any difference in the relative proportions of B and T lymphocytes between infected and non-infected birds (data not shown) but as detailed analysis of T cell subsets was not performed we cannot discount a greater frequency of a subset more responsive to PMA in the infected birds. Given the magnitude of the difference we consider this second possibility less likely. Unfortunately given the paucity of this type of data in this area in avian immunology we have not been able to make extensive direct comparisons, other than to observe that our positive control results are in the range reported by the few directly comparable studies of ELISpot and/or intracellular staining (Ariaans et al., 2008, 2009); however these do not report directly comparable infection data. In the only study regarding the phenotype of responding cells during HPAI infection of chickens (Seo et al, 2002), employing different methods, the percentage of IFNγ producing CD8 positive cells in the spleen was approximately 50% at day 6 post-infection, falling to an average of 15% at 20 days post-infection. This result is much higher than that detected in infected birds in our study; however Seo et al. did not distinguish between IFNγ producing T cells and IFNγ from NK cells, which may account for the difference. We could detect no evidence for NK activation using our method as we were not able to detect a significant number of IFNγ positive cells with splenocytes from non-infected birds cultured with infected CKC (Fig. 4C), or with splenocytes from infected birds cultured with non-infected CKCs (Supplementary Fig. 5). While our study did not identify the TCR subtype of the IFNγ producing CD8 positive cells, it has been hypothesized that the main population involved in IFNγ responses and in viral clearance is TCR αβ (Vβ1, TCR2) (Seo et al., 2002). Interestingly, the control of acute IBV infection has also been attributed to CD8-TCR2 lymphocytes (Collisson et al., 2000). Further studies are required to identify the TCR subsets responsible for the immune response in our model.

Our co-culture method was better able to distinguish responses between infected and control birds than ELISpot using a peptide library. In comparison with recently published work using a high concentration of peptides to analyze influenza-specific responses (Reemers et al., 2012), the co-culture ELISpot is more sensitive and has a significantly lower background. However unlike peptide assays, it lacks precise epitope specificity and cannot distinguish responses against individual proteins. We demonstrated a further level of specificity by infecting CKC with an MVA recombinant virus expressing a fusion protein (NpM1) from a human H3N2 virus (Berthoud et al., 2011). These cells were used to present antigens to splenocytes from birds given a recombinant Fowlpox vaccine, also expressing nucleoprotein and matrix protein 1, and then challenged with a heterologous LPAI virus. Although the NpM1 sequences of the MVA, Fowlpox recombinants and challenge virus were not homologous, these are highly conserved (Lillie et al., 2012) internal influenza antigens (example 98% homology for NP and 100% for M1 protein, Supplementary Fig. 6). We have already shown cross protective immune responses using the MVA vector and the same strain of influenza virus (Boyd et al., 2012). In the current study, we were able to distinguish if individual infected birds were vaccinated or not, since the vaccinated group possessed higher specific responses than unvaccinated birds. Our results suggest that infection of CKC with recombinant virus containing transgenes for an epitope of interest could be used to increase the sensitivity of assays to detect antigen and epitope specific T cells.

In summary we have developed a sensitive method for the detection of antigen specific T cells, which will be important in the analysis of immune responses to both vaccines and pathogens. The assay provides greater sensitivity than the use of inactivated or live virus in ELISpot, and reduced background compared with peptide library ELISpot. Our method is also more accessible to a wider community than methods employing expensive peptide libraries, the interpretation of which data is rendered problematic due to an incomplete knowledge of avian MHC binding specificities. While we have demonstrated its efficacy for influenza, this technique can be applied to the study of T cell responses for many avian pathogens. We also demonstrated that the use of recombinant virus to infect CKC can further define antigen specificity, and additionally reduce the requirement to handle live zoonotic pathogen, an important safety consideration.

## Appendix A Supplementary data

Supplementary Table 1 (aa sequence peptide library): MASQGTKRSYEQ^1^METGGERQNA^2^TEIRASVG^3^RMVGGT*GR^4^FYTQMCTE^5^HKLSDYEG^6^*RLIQNSIP^7^IERMVLSA^8^FDERRNKY^9^LEEHPSAG^10^KDPKKTGG^11^PIYRRRDG^12^KWMRELIL^13^YDKEEIRR^14^IWRQANNG^15^EDATAGLT^16^HLMIWHSN^17^LNDATYQR^20^TRALVRTG^19^MDPRMCSL^20^MQGSTLPR^21^RSGVAGAG^22^VKGVGTMV^23^MELIRMIK^24^RGINDRNF^25^WRGENGRR ^26^TRIAYERM^27^CNILKGKF^28^QTAAQRAM^29^MDQVRESR^30^NPGNAEIE^31^DLIFLARS^32^ALILRGSV^33^AHKSCLPA^34^CVYGLAVA^35^SGYDFERE^36^GYSLVGID^37^PFRLLQNS^38^QVFSLIRP^39^IENPAHKS^40^QLVWMACH^41^SAAFEDLR^42^VSSFIRGT^43^RVVPRGQL^44^STRGVQIA^45^SNENMETM^46^DSSTLELR^47^SRYWAIRT^48^RSGGNTNQ^49^QRASAGQI^50^SVQPTFSV^51^QRNLPFER^52^ATIMAAFT^53^GNTEGRTS^54^DMRTEIIR^55^MMESATPE^56^DVSSQGRG^57^VFELSDEK^58^ATNPIVPS^59^FDMSNEGS^60^YFFGDNAE^61^EYDN^62^. Supplementary Fig. S1. Exogenous antigen presenting cells (APCs) infecting chicken kidney cells (CKCs) with MVA^GFP^/MVA^M1_NP^ could be employed for detection of specific IFN γ responses on splenocytes of infected birds. A) Representative example of chicken kidney cells infected with MVA^GFP^ virus at different MOI and fluorescence was measured by confocal analysis at time points 8, 12 and 24 h after infection. Supplementary Fig. S2. Validation of anti-chicken interferon gamma antibodies by ELISA and ELISpot. The EH9 and AF10 antibodies were tested for their ability to recognize recombinant chicken IFNγ. A) Indirect ELISA to test the specificity of each antibody for recombinant chicken IFNγ. B) An isotype specific ELISA using goat anti-mouse IgG2b as a secondary antibody specific to AF10. C) Sandwich ELISA using EH9 as the coating antibody and AF10 as the detection antibody with either recombinant IFNγ or DEC205. D) Comparison of EH9 and AF10 with commercial antibodies in an ELISpot using ConA stimulated splenocytes (SFU: spot forming units). Supplementary Fig. S3. Analysis of IFN γ ELISpot responses in haplotype B21 birds (Line O) against epitopes of the viral nucleoprotein NP at 1 week postinfection. A) ChIFNγ ELISpot responses of an overlapping peptide library of the viral nucleoprotein (NP). In total, 62 different peptides were pooled in pools of 3 different consecutive peptides and cocultured with splenocytes from infected bird (black, n = 6); control birds (white dot, n = 2). Supplementary Fig. S4. Analysis of IFN-γ CHO transfected cell lines by flow cytometry. EH9-Alexa 647 conjugated antibody and a validated IFNγ Mab80-Alexa 647 permitted to distinguish CHO positive (CHO +) and negative (CHO−) cell lines for IFN-γ production by intracellular analysis. Supplementary Fig. S5. Detection of SPU (spot forming units) at different culture conditions in IFN-γ ELISpot. Influenza infected chicken kidney cells (CKCs) could be used as APCs for the detection IFN- γ responses in influenza infected birds. Representative example of wells from splenocytes from infected (INF) and non-infected (non-INF) birds with culture conditions: influenza infected CKC (CKC^INF^), non-infected CKC (CKC^non-Inf^) and PMA/ionomycin (PMA/Io). Supplementary Fig. S6. Alignment of the NP and M1 protein sequences from recombinant flowpox (NP_FP or M1_FP) and the challenge virus (NP_FP or M1_FP). Conserved residues for the challenge virus are indicated by dots. The sequences are 97.6% and 100% identical for M1 and NP, respectively. The alignment was performed using ClustalW and pairwise comparison using BioEdit (version 7.0.5).
