# Supplementary material for: An infected chicken kidney cell co-culture ELISpot for enhanced detection of T cell responses to avian influenza and vaccination
Source: J Immunol Methods. 2015 Jan;416:40–8. doi: 10.1016/j.jim.2014.10.012 (PMC4334094; doi:10.1016/j.jim.2014.10.012)
Supplement: Supplementary file 1 — Supplementary Table 1 (aa sequence peptide library): MASQGTKRSYEQ1METGGERQNA2TEIRASVG3RMVGGTGR4FYTQMCTE5HKLSDYEG6RLIQNSIP7IERMVLSA8FDERRNKY9LEEHPSAG10KDPKKTGG11PIYRRRDG12KWMRELIL13YDKEEIRR14IWRQANNG15EDATAGLT16HLMIWHSN17LNDATYQR20TRALVRTG19MDPRMCSL20MQGSTLPR21RSGVAGAG22VKGVGTMV23MELIRMIK24RGINDRNF25WRGENGRR 26TRIAYERM27CNILKGKF28QTAAQRAM29MDQVRESR30NPGNAEIE31DLIFLARS32ALILRGSV33AHKSCLPA34CVYGLAVA35SGYDFERE36GYSLVGID37PFRLLQNS38QVFSLIRP39IENPAHKS40QLVWMACH41SAAFEDLR42VSSFIRGT43RVVPRGQL44STRGVQIA45SNENMETM46DSSTLELR47SRYWAIRT48RSGGNTNQ49QRASAGQI50SVQPTFSV51QRNLPFER52ATIMAAFT53GNTEGRTS54DMRTEIIR55MMESATPE56DVSSQGRG57VFELSDEK58ATNPIVPS59FDMSNEGS60YFFGDNAE61EYDN62. Supplementary Fig. S1. Exogenous antigen presenting cells (APCs) infecting chicken kidney cells (CKCs) with MVAGFP/MVAM1_NP could be employed for detection of specific IFN γ responses on splenocytes of infected birds. A) Representative example of chicken kidney cells infected with MVAGFP virus at different MOI and fluorescence was measured by confocal analysis at time points 8, 12 and 24 h after infection. Supplementary Fig. S2. Validation of anti-chicken interferon gamma antibodies by ELISA and ELISpot. The EH9 and AF10 antibodies were tested for their ability to recognize recombinant chicken IFNγ. A) Indirect ELISA to test the specificity of each antibody for recombinant chicken IFNγ. B) An isotype specific ELISA using goat anti-mouse IgG2b as a secondary antibody specific to AF10. C) Sandwich ELISA using EH9 as the coating antibody and AF10 as the detection antibody with either recombinant IFNγ or DEC205. D) Comparison of EH9 and AF10 with commercial antibodies in an ELISpot using ConA stimulated splenocytes (SFU: spot forming units). Supplementary Fig. S3. Analysis of IFN γ ELISpot responses in haplotype B21 birds (Line O) against epitopes of the viral nucleoprotein NP at 1 week postinfection. A) ChIFNγ ELISpot responses of an overlapping peptide library of the viral nucleoprotein (NP). In total, 62 differ [file mmc1.docx]

**Supplementary Table 1 (aa sequence peptide library):**

**MASQGTKRSYEQ^1^METGGERQNA^2^TEIRASVG^3^RMVGGT*GR^4^FYTQMCTE^5^HKLSDYEG^6^*RLIQNSIP^7^IERMVLSA^8^FDERRNKY^9^LEEHPSAG^10^KDPKKTGG^11^PIYRRRDG^12^KWMRELIL^13^YDKEEIRR^14^IWRQANNG^15^EDATAGLT^16^HLMIWHSN^17^LNDATYQR^20^TRALVRTG^19^MDPRMCSL^20^MQGSTLPR^21^RSGVAGAG^22^VKGVGTMV^23^MELIRMIK^24^RGINDRNF^25^WRGENGRR^26^TRIAYERM^27^CNILKGKF^28^QTAAQRAM^29^MDQVRESR^30^NPGNAEIE^31^DLIFLARS^32^ALILRGSV^33^AHKSCLPA^34^CVYGLAVA^35^SGYDFERE^36^GYSLVGID^37^PFRLLQNS^38^QVFSLIRP^39^IENPAHKS^40^QLVWMACH^41^SAAFEDLR^42^VSSFIRGT^43^RVVPRGQL^44^STRGVQIA^45^SNENMETM^46^DSSTLELR^47^SRYWAIRT^48^RSGGNTNQ^49^QRASAGQI^50^SVQPTFSV^51^QRNLPFER^52^ATIMAAFT^53^GNTEGRTS^54^DMRTEIIR^55^MMESATPE^56^DVSSQGRG^57^VFELSDEK^58^ATNPIVPS^59^FDMSNEGS^60^YFFGDNAE^61^EYDN^62^**


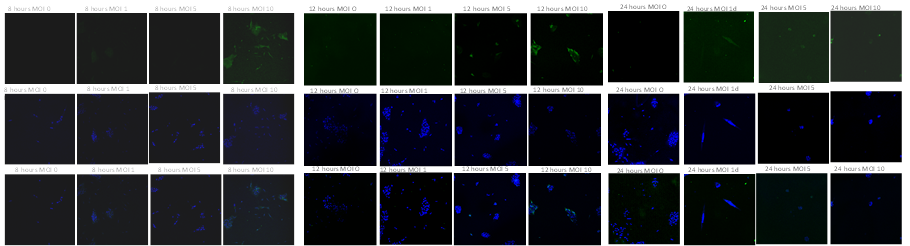


**Supplementary Figure S1. Exogenous antigen presenting cells (APCs) infecting chicken kidney cells (CKCs) with MVA^GFP^/ MVA^M1_NP^** **could be employed for detection of specific IFN γ responses on splenocytes of infected birds**. A) Representative example of chicken kidney cells infected with MVA^GFP^ virus at different MOI and fluorescence was measured by confocal analysis at time point 8, 12 and 24 hours after infection.

###

### Supplementary Figure S2. Validation of anti-chicken interferon gamma antibodies by ELISA and ELISpot.

The EH9 and AF10 antibodies were tested for their ability to recognize recombinant chicken IFNγ. A) Indirect ELISA to test the specificity of each antibody for recombinant chicken IFNγ. B) An isotype specific ELISA using goat anti-mouse IgG2b as a secondary antibody specific to AF10. C) Sandwich ELISA using EH9 as the coating antibody and AF10 as the detection antibody with either recombinant IFNγ or DEC205 D) Comparison of EH9 and AF10 with commercial antibodies in an ELISpot using ConA stimulated splenocytes (SFU: spot forming units).

**Supplementary Figure S3. Analysis of IFN γ Elispot responses in haplotype B21 birds (line O) against epitopes of the viral nucleoprotein NP at 1 weeks postinfection**. A) chIFNγ Elispot responses of an overlapping peptide library of the viral nucleoprotein (NP). In total, 62 different peptides were pooled in pools of 3 different consecutive peptides and cocultured with splenocytes from infected bird (black, n=6); Control birds (white dot, n=2).


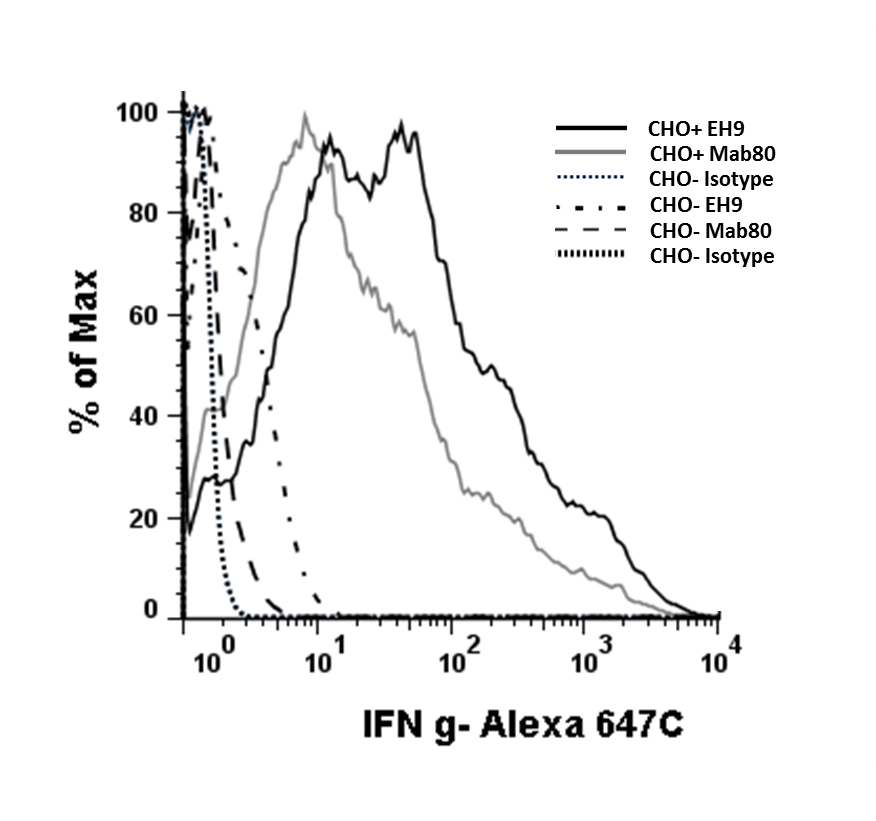


**Supplementary Figure S4. Analysis of IFN-γ CHO transfected cells lines by flow cytometry**. EH9-Alexa 647 conjugated antibody, and a validated IFNγ Mab80-Alexa 647 permitted to distinguish CHO positive (CHO+) and negative ( CHO-) cell lines for IFN-γ production by intracellular analysis.

***
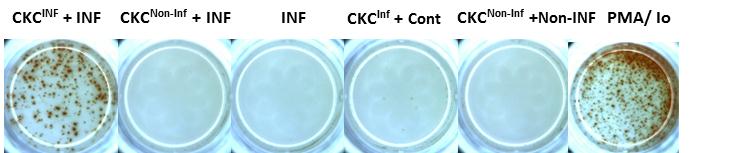
***

**Supplemental Figure S5. Detection of SPU (Spot forming units) at different culture conditions in IFN- γ Elispot.** Influenza Infected Chicken Kidney cells (CKCs) could be used as APC for the detection IFN- γ responses in influenza infected birds. Representative example of wells from splenocytes from infected (INF) and non-infected bird (Non-INF) with culture conditions: Influenza infected CKC (CKC^INF^), non infected CKC (CKC^Non-Inf^) and PMA/Ionomycin (PMA/Io).


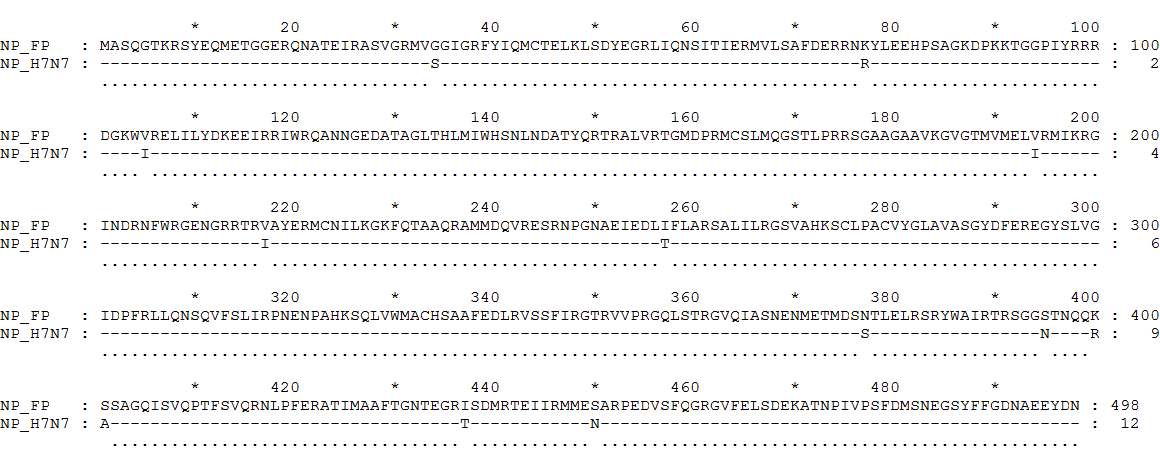


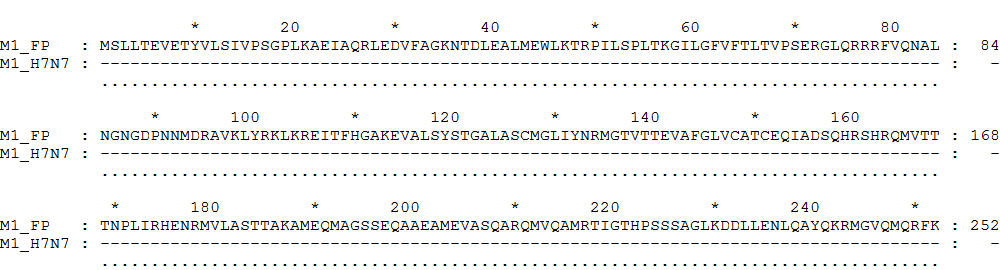


**Supplementary Figure S6. Alignment of the NP and M1 protein sequences from recombinant flowpox (NP_FP or M1_FP) and the challenge virus (NP_FP or M1_FP).** Conserved residues for the challenge virus are indicated by dots. The sequences are 97.6% and 100% identical for M1 and NP respectively. The alignment was performed using ClustalW and pairwise comparison using BioEdit (version 7.0.5)
